# *Clostridium ramosum* rapidly identified by MALDI-TOF MS. A rare gram-variable agent of bacteraemia.

**DOI:** 10.1099/acmi.0.000137

**Published:** 2020-06-15

**Authors:** M.C. Legaria, S.D. García, V. Tudanca, C. Barberis, L. Cipolla, L. Cornet, A.M.R. Famiglietti, D. Stecher, C.A. Vay

**Affiliations:** ^1^​ Universidad de Buenos Aires, Facultad de Farmacia y Bioquímica, Hospital de Clínicas José de San Martín, Departamento de Bioquímica Clínica, Cátedra de Microbiología Clínica, Buenos Aires, Argentina; ^2^​ Universidad de Buenos Aires, Facultad de Medicina, Hospital de Clínicas José de San Martín, Servicio de Infectología, Buenos Aires, Argentina; ^3^​ Servicio Bacteriología Especial, INEI-ANLIS "Dr. Carlos G. Malbrán", Buenos Aires, Argentina

**Keywords:** *Clostridium ramosum*, Bacteraemia, MALDI-TOF MS, *Erysipelatoclostridium ramosum*, Infection, Antimicrobial susceptibility

## Abstract

*
Clostridium ramosum* is an enteric anaerobic, endospore-forming, gram-positive rod with a low GC content that is rarely associated with disease in humans. We present a case of *C. ramosum* bacteraemia. To the best of our knowledge, this is the second case of *C. ramosum* bacteraemia in an elderly patient presenting with fever, abdominal pain and bilious emesis. We highlight the Gram stain variability, the lack of visualization of spores and the atypical morphology of the colonies that showed *C. ramosum* in a polymicrobial presentation that initially appeared to show monomicrobial bacteraemia. The microorganism was rapidly identified by matrix-assisted laser desorption/ionization time-of-flight mass spectrometry (MALDI-TOF MS). We present a comprehensive literature review of 32 cases of clinical infections by *C. ramosum* in which we describe, if available, sex, age, clinical symptoms, predisposing conditions, other organisms present in the blood culture, other samples with *C. ramosum *, identification methodology, treatment and outcome.

## Introduction

*Clostridium ramosum* is an anaerobic, endospore-forming and gram-positive rod that is an inhabitant of the gastrointestinal tract [1]. The organism was renamed *Erysipelatoclostridium ramosum* in 2013 [2]. Routine diagnostic laboratories may have difficulty in identifying *C. ramosum* [3].

*Clostridium* bacteraemia (CB) account for approximately 1 % of all significant blood culture isolates. Most are caused by *Clostridium perfringens* [4, 5]. Occasionally, *C. ramosum* has been found in association with bacteraemia and other infections sites [6, 7].

We report a case of *C. ramosum* bacteraemia (CRB) with a devastating outcome in an elderly patient with rheumatoid arthritis who was under long-term steroid treatment, with apparent abdominal origin. The purpose of this report was:

- to alert microbiologists to suspect aerobic–anaerobic poly-microbial bacteraemia when gram-variable rods with apparent monomicrobial origin are observed in anaerobic bottles;
- to show the advantages of matrix-assisted laser desorption/ionization time-of-flight mass spectrometry (MALDI-TOF MS) for fast and reliable identification of *C. ramosum *;
- to present a literature review of bacteraemia and other clinical infections caused by *C. ramosum *.

## Case Presentation

A patient in their 90s with a medical history of anaemia, bedridden status and rheumatoid arthritis under prednisone treatment was admitted to the emergency room with fever up 38.5 °C, bilious emesis and abdominal pain. Abdominal tenderness, diminished bowel sounds and pain at abdominal examination were observed. Heart murmurs were hypophonic. The patient was in poor general condition, with a tendency to sleep, and confused. Blood pressure was 70/40 mmHg and the respiratory rate was 30 min^−1^. Laboratory findings showed a white blood cell count of 12×10^9^ l^−1^ (85 % neutrophils), platelets 310×10^9^ l^−1^, glucose 66 mg dl^−1^, urea 63 mg dl^−1^, creatinine 1.33 mg dl^−1^, Na^+^ 123 meq l^−1^, K^+^7 meq l^−1^, Cl^−^ 83 meq l^−1^, aspartate aminotransferase 27 IU l^−1^, alanine aminotransferase 10 IU l^−1^, alkaline phosphatase 65 IU l^−1^, total bilirubin of 1.20 mg dl^−1^ and serum lactate 5.2 mmol l^−1^. Urine and blood cultures were drawn. Because aerobic bottles were not available at that time in the emergency room, and the antimicrobial and surgical treatment had to be implemented urgently, two anaerobic bottles were collected through two venipuncture (10 ml of blood in each vial) from different sites. Piperacillin/tazobactam (4.5 g every 6 h, IV) treatment was started. Because the patient was in poor general condition, and because of the severity of the presentation, no images were taken. As bowel perforation was suspected, an exploratory laparotomy was going to be performed. Unfortunately, the patient died just before the procedure was begun. No autopsy was performed.

**Fig. 1.**
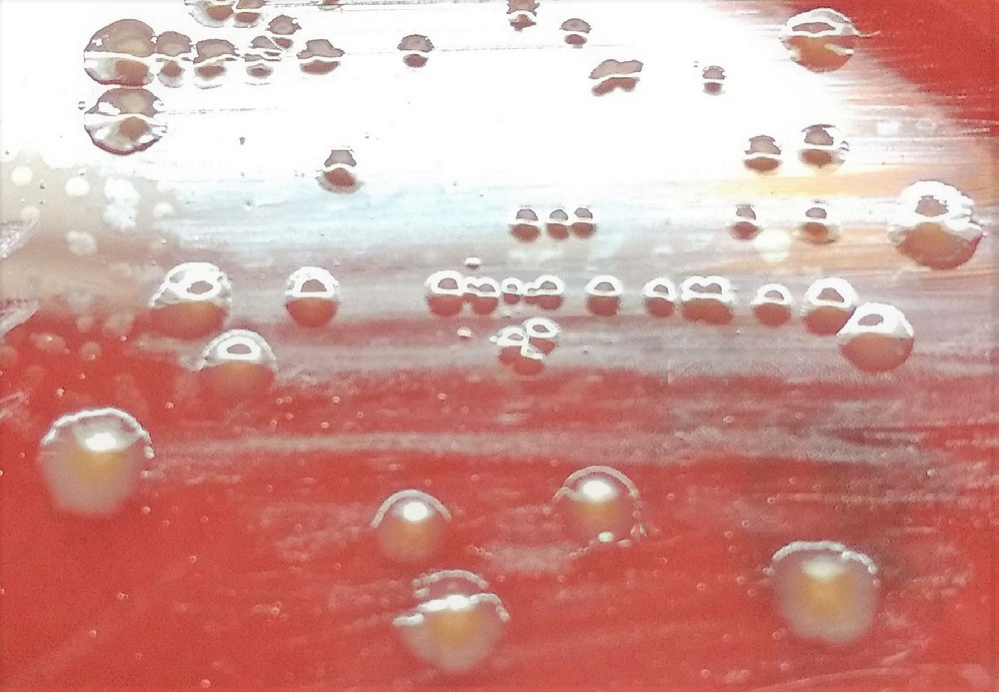
*
C. ramosum* colonies on Brucella blood agar.

**Fig. 2.**
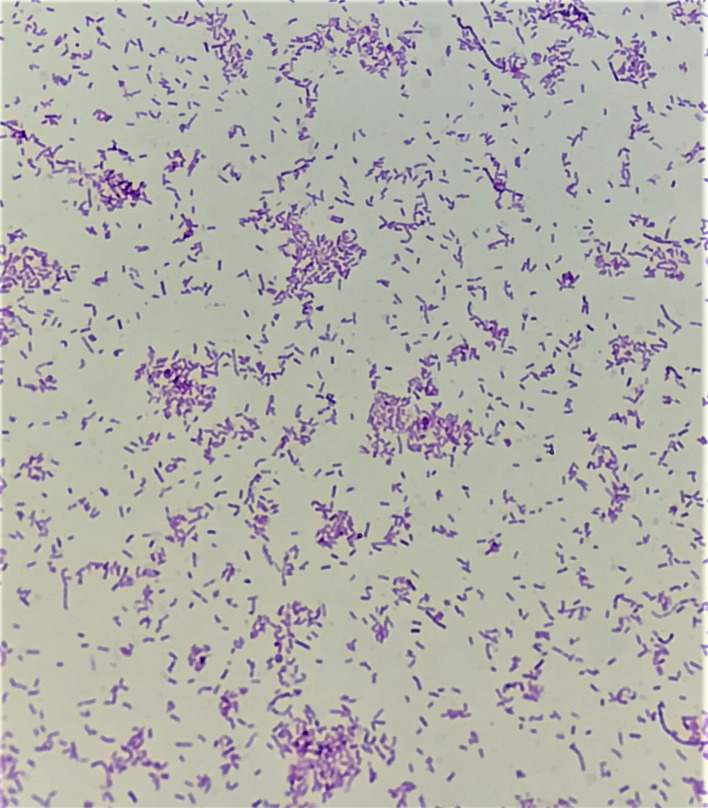
*
C. ramosum* Gram stain.

Urine culture was negative. Blood samples were cultured using the BD BACTEC FX system (Becton Dickinson, Franklin Lakes, NJ, USA). After a 3 h incubation period, one of the anaerobic bottles (bottle 1) became positive and Gram staining demonstrated gram-variable/negative rods. Because an aerobic subculture yielded growth of *Escherichia coli *, an anaerobic culture was not performed. After a 4 days and 7 h of incubation, the other anaerobic bottle (bottle 2) showed a thin gram-negative rod. The organism did not grow aerobically, and thus an anaerobic subculture was performed. Anaerobic growth on Brucella blood agar supplemented with hemin and vitamin K (BA) yielded a pure culture of small, greyish, smooth, round, non-haemolytic colonies with whole edges after 48 h that turned brown with longer incubation (Fig. 1). The isolate was identified as *Clostridium ramosum* by the MALDI-TOF Microflex Biotyper 3.1 with a score >2. (Bruker-Daltonics) and by conventional methods [8]. The organism stained gram-variable, although it demonstrated susceptibility to vancomycin (5 µg) and resistance to colistin (10 µg) special potency identification discs, suggesting that it was a gram-positive bacterium (Fig. 2). The isolate was nonmotile. Tests for catalase, indole, lecithinase, lipase, gelatin hydrolysis, nitrate, and the reverse CAMP test, were negative. The isolate was able to ferment maltose, glucose, sucrose, fructose, mannose, melibiose, cellobiose, raffinose, salicin, lactose and mannitol, but not arabinose and xylose. Bile esculin agar showed enhanced growth with hydrolysis of esculin.

Given that *C. ramosum* could stain as a gram-negative rod, we decided to re-examine bottle 1 under the suspicion of a polymicrobial bacteraemia. Hence, in order to find out if *C. ramosum* was present we sub-cultured it anaerobically onto BA, Brucella supplemented with vitamin K, hemin and amikacin (BAA), and BAA supplemented with vancomycin (BAAV). *C. ramosum* grew on the BA and BAA after 48 h of incubation; BA yielded *E. coli* too. Attempts to grow organisms on BAAV failed.

In order to confirm species identification, PCR amplification of the 16S rRNA was performed. The nearly complete sequence of the 16S rRNA gene was amplified by PCR with the conserved primers 8F (5′-AGAGTTTGATYMTGGCTCAG-3′) and 1942R (5′-ACCTTGTTACGACTT-3′) as described previously [9]. Cycling sequencing was performed using the Big Dye Terminator v3.1 Cycle Sequencing kit (Applied Biosystems) and the ABI PRISM 3100 Genetic Analyzer (PE Applied Biosystems). The sequence obtained for the isolate analysed was assembled, manually corrected and compared to publicly available sequences in GenBank using the Basic Local Alignment Search Tool (blast) algorithm of the National Center for Biotechnology Information (NCBI). Alignments were performed by a hierarchical multiple alignment method implemented in the program clustal x. The sequence obtained showed 100 % identity with the sequence corresponding to the 16S RNA ribosomal gene of *C. ramosum* type strain JCM 1298 (GenBank accession no. AB595128).

*E. coli* was susceptible to piperacillin/tazobactam. *C. ramosum* did not produce beta-lactamase [nitrocefin disc (bioMerieux)]. Antibiotic susceptibilities were determined by E-test (bioMérieux), and were interpreted using the Clinical and Laboratory Standards Institute (CLSI) M11-A8 document [10]. *C. ramosum* showed sensitivity to ampicillin (MIC 0.016 µg ml^−1^), piperacillin/tazobactam (MIC 0.032 µg ml^−1^), cefoxitin (MIC 4 µg ml^−1^), metronidazole (MIC 0.094 µg ml^−1^), imipenem (MIC 0.025 µg ml^−1^) and clindamycin (MIC 0.075 µg ml^−1^).

## Discussion and Review of Literature

*C. ramosum* is one of the *Clostridium* spp. that have been reported to stain gram-negative despite having a typical gram-positive cell wall [11]. In an extensive update of clostridial classification, more than 50 bacteria, previously placed in the genus *Clostridium,* have been reassigned to other taxonomic groups [12, 13]. However, many organisms still retained the *Clostridium* name, causing major confusion in clostridial taxonomy [12, 14, 15]. *C. ramosum *, a member of the cluster XVIII, along with three other *Clostridium* species, was transferred by Ludwig *et al*. in 2009 to the family *Erysipelotrichaceae* in the class *Erysipelotrichi* [12]. It has been proposed to assign *C. ramosum* and four related species to the new genus *Erysipelatoclostridium* [2, 15]. The microorganism was renamed *Erysipelatoclostridium ramosum* in 2013. The genus *Erysipelatoclostridium* is in the new family *Erysipelatoclostridiaceae* with other members of the XVIII clostridial cluster and was recently confirmed as a monophyletic group in a standardized revision of the taxonomy [2, 16].

The isolation and identification of *C. ramosum* in a polymicrobial presentation has some difficulties. *C. ramosum* can easily be misidentified or overlooked because it usually stains as a gram-negative rod and its terminal spores are hard to demonstrate [3, 17–19].

The Gram stain may be the oldest and most entrenched technique still in use in the microbiology laboratory. It is well recognized that certain gram-positive species often stain gram-negative or gram-variable as cultures age because of cell wall changes with loss of viability [20]. Although most *Clostridium* species are anaerobic, endospore-forming and gram-positive rods, some species may be stained gram-variable or gram-negative and their spores could be difficult to detect. Some of these microorganisms belong to the so-called ‘RIC group’: *C. ramosum, Clostridium innocuum* and *Clostridium clostridioforme* [3]. *C. ramosum* may be difficult to recognize in the laboratory because of its Gram stain variability and lack of spores, and the atypical clostridial morphology of its colonies [3, 17–19, 21]. It was reported that the loss of gram-positive appearance occurs most frequently in direct stains of clinical material, or in cultures after incubation for extended periods, or in species showing terminal spores [3, 22]. Therefore, *C. ramosum* can be misidentified as belonging to other genera, so the number of positive cultures is probably underestimated [23].

In our case, the organism presented as thin gram-negative rods, which made microbiological interpretation difficult. In fact, an anaerobic sub-culture was initially not carried out for bottle 1, because it was interpreted as showing monomicrobial growth, i.e. *E. coli *. Physicians and microbiologists should maintain a high level of awareness because the Gram stain of a blood culture can be misinterpreted [17, 24].

Nowadays, several commercially available identification techniques, ranging from enzyme-based systems to newer molecular PCR-based techniques (such as 16S rRNA sequencing) and MALDI-TOF MS, are in use. However, most of the kits for biochemical identification of *C. ramosum* need to be supplemented with additional tests for the complete identification of the organism [3, 18, 25, 26]. Further, ANC cards (VITEK 2, bioMérieux, France) have identified *C. ramosum* as *C. clostridioforme* [27]. For all these reasons, the 16S rRNA gene needs to be sequenced to confirm the identification [1, 9]. These factors probably lead to underreporting of this clostridial species in human infections [28].

For diagnosis and treatment purposes it is important to ensure fast and accurate identification of *Clostridium* species. The current trend is for instrumental methods to replace traditional microscopic and biochemical investigations for rapid identification. However, 16S rRNA gene sequencing is time-consuming, which may inhibit this possibly becoming a routine approach. Modern alternatives such as MALDI-TOF MS offer a fast, accurate, sensitive, robust and inexpensive solution for routine characterization and typing of microbes in laboratories. In fact, both VITEK MS (VITEK MS database 2.0, bioMérieux, France) and MALDI-TOF MS (Bruker Biotyper – Becton Dickinson) showed excellent performance for the identification of *C. ramosum* [27, 29]. However, it is important to consider which database has been used in the identification process, because in a study carried out by Lee *et al*., 2/7 *C *. *ramosum* isolates were not identified by VITEK MS with the IVD 1.1 database [30]. Fortunately, this was updated to the VITEK MS database 2.0, and interestingly 7/7 *C *. *ramosum* strains included in a recently published study were identified correctly [27].

CRB particularly occurs in adults predisposed to infection because of underlying malignancy and immunosuppression, and patients with bowel perforation and abscess formation [6, 31, 32], but also in immunocompetent patients [28]. CRB has comprised 0–13 % of CB in published retrospective case series [4, 33]. The medical literature contains a paucity of CRB documented cases (Table 1) [6, 7, 23, 28, 31, 32, 34–42].

**Table 1.** Reported cases of *C. ramosum* infections

No., year reported, reference, country	Sex/age (years)	Clinical symptoms	Predisposing condition	BC/CRBC/other organisms in the BC	Others samples with ***C. ramosum***/other microorganisms in samples with *C. ramosum* (except BC)/***C. ramosum*** identification methods	Antimicrobial treatment	Outcome/BC control
1,1979, [47], USA	M/2	Fever, otalgia. History of fussiness,	nr	No/-/–	Middle ear exudate/group A beta haemolytic streptococci/Conventional	AMP	Survived/NR
2, 1983, [34], USA	M/53	Chills, fever (38 °C), backache	Renal transplant recipient, prednisone and azathioprine treatment, bleeding duodenal ulcer, diverticulosis, periodontal disease.	Yes/Yes/No	Left femoral artery emboli/No/nr	CLIN/PEN	Fatal/Neg
3, 1988, [46], USA	F/28	Fever (39 °C), fatigue, abdominal pain, nausea, vomiting (coffee ground emesis), melena	One week history of blunt abdominal trauma, dark urine for 1 month	Yes/No/* Bacteroides fragilis *	Perinephric abscess/*Bacteroides fragilis Peptostreptococcus* sp./nr	AMP,+CLIN+GEN	Survived/NR (nephrectomy)
4 1991 [4] USA	F/26	Fever (40 °C)	Colorectal tumour, necrotic tumour	Yes/Yes/* Bacteroides fragilis *	No/–/nr	nr	Survived/nr
5 1991, [4], USA	M/79	Fever (40 °C)	Fever unknown origin, colorectal tumour	Yes/Yes/* Clostridium innocuum *	No/–/nr﻿ ﻿﻿﻿NR	nr	Survived/nr
6, 1995, [31], USA	F/3	nr	Sepsis, perforated viscus	Yes/Yes/No	No/–/API anaerobic SystemNR	PEN	Survived/nr
7, 1995, [31], USA	M/3	nr	Acute otitis media	No/–/-	Middle ear/No/API anaerobic System	AMX	Survived/NR
8, 1998 [19], Spain	M/59	Fever (37.5 °C), nausea, bilious emesis, headache	HTN, alcohol abuse, generalized tonic-clonic seizure, diffuse bronchiectasis, gout	No/–/-	Brain abscess/* Peptostreptococcus* sp./ nr	PEN	Survived/NR
9, 1999, [43], The Netherlands	F/11	Fever (40.5 °C), chills, myalgia, loss of appetite, watery bloody diarrhoea	Acute lymphatic leukaemia, mucositis, gas gangrene in the neck and thorax, chemotherapy, oral mucositis, gas gangrene in the neck and thorax	Yes/Yes/*Candida albicans*	No/–/Conventional	VAN+GEN+CAZ	Fatal/nr
10, 2001,[21], India	M/5	Fever 1 ½ month, irritability, headache, two previous tonic convulsions	History of bilateral chronic suppurated otitis media	No/-/–	Cerebellar abscess/No/Conventional	CHLO, MTZ, AMP	Survived/nr
11, 2001, [35], USA	F/91	Fever (39 °C)	HTN, DM, nursing home resident, bedridden, demented, multiple pressure sores, stage III/ IV ulcer in left hip and sacrum (foul smelling discharge/necrotic/gas), osteomyelitis	Yes/Yes/No	No/–/API 20A	AMS+GEN	Survived/Neg
12, 2003, [18], France	M/74	Afebrile, severe lower lumbar pain (6 weeks), functional incapacity	Weight loss of 9 kg, prostatic adenoma	Yes/No/No	Paravertebral abscess/No/API 20A (*C. ramosum-Actinomyces israelii*), 16S rRNA gene sequencing	AMX+ CIP, AMX+MTZ	Survived/nr
13, 2005, [45], Turkey	M/7	Headache, vomiting	Intracranial hydatid cyst, *Echinococcus granulosus.* Pleural empyema	No/–/-	Cystic component/No/nr	MER	Survived/NR
14, 2006, [36], USA	M/49	Fever., weight loss	DM, lung abscess.	Yes/Yes/No			
15, 2008, [7], USA	M/60	Fever, headache	Acute myeloid leukaemia, vesicula skin lesion on his back	Yes/Yes/No	No/–/nr	VAN+CAZ, AMS	Survived/Neg
16, 2008, [38], France	M/72	Fever	Aortic prosthesis, endocarditis	Yes/Yes/No	No/–/16S rRNA gene sequencing	PTZ, AMX	Survived/nr
17, 2012, [23], Spain	F/79	Fever (39 °C), abdominal pain, watery diarrhoea, weakness.	Immunocompetent, previous treatment with CIP and AMC, colonic ulcers	Yes/Yes/No	No/–/nr	MTZ	Survived/nr
18, 2014, [32], USA	F/80	Fever (38.6 °C), abdominal pain, nausea, bilious emesis	History of endometrial cancer, hysterectomy. Pancreatic adenocarcinoma, pancreatectomy, splenectomy. DM, small bowel obstruction, perforated sigmoid colon, multiple diverticula.	Yes/Yes/* Bacteroides fragilis *	No/–/nr	PTZ/ERT	Fatal/Neg
19, 2015, [37], Korea	F/89	Fever (38.2 °C), systemic weakness	Ulcers in ankle, and coccyx	Yes/Yes/No	No/–/Vitek II ANC card (bioMérieux, Inc.), 16S rRNA gene sequencing, MALDI-TOF MS VITEK MS (bioMérieux, Inc.)	CRO, TIG	Survived/Neg
20, 2015, [37], Korea	M/70	Fever (38.1 °C), abdominal pain	Bile duct stones, colon cancer, liver and peritoneal metastases	Yes/Yes/No	No/–/Vitek II ANC card (bioMérieux, Inc.), 16S rRNA gene sequencing, MALDI-TOF MSVITEK MS (bioMérieux, Inc.)	CIP, MER+TEI	Survived/nr
21, 2015, [28], USA	M/26	Low-grade fever, dull ache in the left leg, purulent drainage of the left tibia wound sinus tract	Immunocompetent, motor vehicle crash, left open tibia shaft fracture, intramedullary nailing of the tibia, locking screws, multiple debridements and surgeries, osteomyelitis	Yes/Yes/MR *Staphylococcus aureus *	Surgical wound (tibia after hardware removal) /No/nr	TMP/SMX+AMS/DAP+ERT+MTZ/ VAN+TIG	Survived/nr (amputation)
22, 2016 [39] Spain	F/62	6-months of pain in her left prosthetic knee. Fever	Rheumatoid arthritis, methotrexate and prednisone treatment	Yes/No/No	Synovial fluid, intraoperative cultures (surgical debridement)/No/No	VAN+CAZ, CIP+LNZ, MER+MTZ, PEN+CLIN, MTZ	Fatal/NR
23, 2016, [39], Spain	M/53	Fever, pain in left hip	Osteoarthritis of the hip, prosthetic aortic valve, chronic hepatitis C infection, chronic renal failure on dialysis, abscess left wrist, girdlstone resection arthroplasty	Yes/Yes/No	Abscess left wrist, left hip intraoperative sample/No/DNA sequencing	VAN+GEN, AMP+GEN, AMC	Fatal/nr
24–28 2017 [6] USA	1- F/81	4/5 patients fever	Refractory acute myeloid leukaemia. Pneumibilis.	Yes/Yes/* Acinetobacter* sp.	No/–/ RapID ANA II System, Remel. 16S rRNA gene sequencing.	CEF	Fatal/nr
	2- F/66		Pre-β cell acute lymphoid leukaemia. Dilated cmmon bile duct.	Yes/Yes/no	No/-/ RapID ANA II System Remel. 16S rRNA gene sequencing.	CEF+MTZ	Survived/nr
	3 -M/79		Acute lymphoblastic leukaemia	Yes/Yes/no	No/-/RapID ANA II System, Remel. 16S rRNA gene sequencing.	CAZ+MTZ	Fatal/nr
	4 -M/49		Cirrhosis hepatic, encephalopathy, CDI	Yes/Yes/* Citrobacte *r sp.	No/-/ RapID ANA II System, Remel. 16S rRNA gene sequencing.	CEF	Fatal/nr
	5 -F/83		Refractory acute myeloid leukemia, CDI	Yes/Yes/no	No/-/RapID ANA II System, Remel. 16S rRNA gene sequencing.	CEF	Survived/nr
29,2018, [44] Japan	F/44	Fever (37.4 °C), pain in the genital area (6 weeks), fatigue, loss of appetite	Central DBT insipidus, Insulin-dependent DBT mellitus, frequent hospital, admissions (6 weeks), undernutrition, pancreatectomy, splenectomy, cholecystectomy, incontinence, urinary catheter, edema of the pelvis and femoral areas	Yes/Yes/* Streptococcus constellatus *	No/-/16S rRNA gene sequencing	GEN,CRO,MER+CLIN+VAN	Fatal/NR
30, 2018, [17], Switzerland	M/74	Abdominal pain, diarrhoea, confused, low blood pressure	Alcoholic liver cirrhosis, peritoneal dialysis	No/–/-	Peritoneal fluid/No/nr	CFZ+CAZ/VAN	Comfort care/NR
31, 2019, [48], Switzerland	F/26	Pain at the right distal femur	Metastatic Ewing’s sarcoma, chemotherapy, amputation, tumour resection, progressive resorption of the allograft (right distal femur)	No/–/-	Bone and allograft biopsies/No/MALDI-TOF, 16S rRNA gene sequencing	CLIN	Survived/NR
32, 2019, [49], Japan	M/73	Upper abdominal pain, fever	Acute aortic dissection, ulcer-like projection, laryngeal nerve paralysis, diverticula in ceccum and colon	Yes/No/No	Aortic wall and surrounding tissue/No/nr	MER+MTZ/MIN	Survived/NR
33, 2019, present case, Argentina	nr/~90s	Fever (38 °C), abdominal pain, bilious emesis	HTN, rheumatoid arthritis, corticoids, chronic anaemia	Yes/Yes/* Escherichia coli *	No/–/MALDI-TOF, 16S rRNA gene sequencing, conventional	PTZ	Fatal/ND

CRBC, *C. ramosum* in blood culture; BC, blood culture; GI, gastrointestinal tract; IV, intravenous; DM, diabetes mellitus; HTN, hypertension; nr, not reported; Neg, negative; API 20A, API 20A anaerobic strip (bioMérieux, Hazelwood, MO); ND: not drawn. PEN, penicillin, AMP, ampicillin; AMS, ampicillin sulbactam; AMX, amoxicillin; AMC, amoxicillin/clavulanic acid; GEN, gentamicin; VAN, vancomycin; PTZ, piperacillin/tazobactam; CIP, ciprofloxacin; ERT, ertapenem; MER, meropenem; TEI, teicoplanin; MTZ, metronidazole; CRO, ceftriaxone; CEF, cefepime; TIG, tigecycline; CFZ, cefazolin; CLIN, clindamycin; CAZ, ceftazidime; CHLO, chloramphenicol; DAP, daptomycin; TMP/SMX, trimethoprim/sulpamethoxazole; LNZ, linezolid; CDI, *Clostridioides difficile* infection.

*C. ramosum* has been reported to cause osteomyelitis, septic arthritis, mastoiditis, spondylodiscitis, otitis media, pyelonephritis, septic arterial emboli, endocarditis, gas gangrene, septic pseudoarthrosis, peritoneal dialysis-related peritonitis, liver abscess, brain abscess, cerebellar abscess, lung abscess, Fournier’s gangrene, pseudomembranous colitis, infected thoracic aortic aneurysm and infection of intracranial hydatid cyst (Table 1) [17–19, 21, 23, 28, 34, 36, 38, 39, 43–49]. Most *C. ramosum* isolates were obtained from blood culture, and occasionally from other samples, such as:

(1) Abscess in a case of cerebellar abscess secondary to otitis media in a 5-year-old boy with bilateral chronic suppurative otitis media for 4 years for which he was being treated with no response [21]. (2) Prosthetic material in a case of septic pseudarthrosis after an orthopaedic procedure with an allograft in a young female patient with Ewing’s sarcoma [48]. (3) Peritoneal fluid in a case of peritoneal dialysis-related peritonitis. Peritoneal fluid was cultivated in a set of BacT/ALERT FAN Plus aerobic and anaerobic blood culture bottles (bioMérieux, Marcy-l’Étoile, France). *C. ramosum* could be detected within 1 day in the anaerobic bottle [17]. (4) Wound in a case of osteomyelitis in an immunocompetent patient after traumatic injury [28]. (5) Perinephric abscess in a case of emphysematous pyelonephritis [46]. (6) Paravertebral abscess in a case of spondylodiscitis [18]. (7) Left femoral artery emboli in a case of endocarditis [34]. (8) Aortic wall and surrounding tissue in a case of infected thoracic aortic aneurysm [49]. (9) Middle ear in two cases of acute otitis media [31, 47]. (10) Cystic component in a case of intracranial hydatid cyst infection [45]. (11) Abscess left wrist and left hip intraoperative sample in a case of osteoarthritis of the hip [39]. (12) Abscess in a case of brain abscess [19]. (13) Synovial fluid and intraoperative samples in a case of knee prosthetic joint infection [39]. Interestingly, 11 of these 14 samples (78 %) yielded *C. ramosum* in pure culture [17, 18, 21, 28, 31, 34, 39, 45, 48, 49].

CB frequently has a polymicrobial presentation [50]. Multiple-organism bacteraemia is a sign of intra-peritoneal sepsis [51]. *C. ramosum* is an inhabitant of the intestinal tract, and has only rarely been associated with severe infections [52–54]. Like other series, in our patient, we suspect an abdominal source because of the clinical and polymicrobial presentation with an enteric bacterium, i.e. *E coli* [55]. Although *C. ramosum* is non-pathogenic, it is able to produce immunoglobulin A1 and A2 proteases, which may facilitate intestinal mucosal penetration, allowing the bacterium to escape the mucosal defense in particularly susceptible patients [53, 54, 56, 57]. Because the GC% of the gene encoding this enzyme is significantly higher than the entire genome of *C. ramosum *, it seems that this gene was acquired recently through horizontal gene transfer [58]. Moreover, only a fraction of the isolates are IgA protease-positive, suggesting that this enzyme is not essential to the bacterium [53] Although IgA1 is predominant in the upper respiratory tract (URT), equal proportions of IgA1 and IgA2 occur in the intestine. Most bacteria producing IgA1 protease activity colonize the URT, whereas *C. ramosum* is a natural inhabitant of the intestine. Therefore, possession of a proteinase cleaving both molecules seems to be beneficial for this microorganism and its *in vivo* fitness in the colonization of the intestine mucosa [53, 57]

On the other hand, it is interesting to note the growing importance of *C. ramosum* in other diseases apart from the infectious states. The gut microbiome is currently an area of intensive research. In recent studies, *C. ramosum* has been associated with obesity, diabetes, malignancy, enterohemorrhagic *E. coli* (EHEC) infection, and attention has also been paid to its use as an immunization treatment or as a biomarker for cancer [58–66].

Most reported CRB cases, like ours, occur in adults predisposed to infection because of underlying malignancy or immunosuppression, and patients with bowel perforation and abscess formation [6, 31, 32] (Table 1).

Advanced age increases the risk of clostridial infection, independent of comorbidities, which could be explained by age-related increase of clostridial species in the normal intestinal microbiota [41]. Moreover, as in our case, CRB can be associated with high mortality [6]. In one CB study, *C. ramosum* was among the three most highly placed isolates in the case fatality rates [41]. It is notably that 9/22 (41 %) reported CRB cases had a fatal outcome [of these, 6/9 (66 %) had polymicrobial bacteraemia], while 9/22 (41 %) were polymicrobial. Of 31 cases of *C. ramosum* infections with available data, 26 (84 %) presented fever. Only 1/11 patients who did not present CRB had a fatal outcome (Table 1).

The clinical significance of *Clostridium* isolated from blood culture is a subject of ongoing debate. Some believe this organism should never be dismissed as a contaminant [67, 68]. Others have demonstrated that *Clostridium* species are usually non-pathogenic [69, 70]. However, it is important to consider that microbes recovered from blood cultures should be assumed to be significant if they have established pathogenic potential or are present, as in our case, in two blood cultures [71]. Unfortunately, an anaerobic subculture was not performed for bottle 1 [* E. coli* and *C. ramosum *, time-to-positivity (TTP) 3 h] at the time it became positive. With regard to bottle 2 (* C. ramosum *, TTP 4 days and 7 h), a previous study demonstrated that most (85 %) positive signals on days 3 to 5 were ultimately disregarded as contamination, but, late positive bottles, as in our case, can play a valuable role in the interpretation of culture, mainly if two blood cultures yielded *C. ramosum* [72]. Because of the patient’s predisposition to a clostridial infection, the clinical presentation and outcome, and the fact that two separate blood cultures obtained from different sites (two venipuncture) were positive for *C. ramosum *, a rare microorganism not usually found as contaminant, we could assume that CRB in our patient was clinically significant.

On the other hand, it is important to consider that a short TTP has been reported to be an independent predictor of fatal outcome in patients with diverse-source *E. coli* bacteraemia [73]. Previous studies have shown that the TTP is shorter from patients whose *E. coli* bacteraemia are of unknown origin or non-urinary tract, from females, from cases of shock or severe sepsis, or from patients who died. Indeed, *E. coli* bacteraemia was an independent predictor of fatal outcome in patients with diverse-source bacteraemia [74, 75]. TTP and the clinical relevance of the individual pathogens of the polymicrobial culture cannot be determined or is difficult to determine [75]. One study revealed that by 48 h, 98 % of aerobic gram-positive and gram-negative bloodstream infections were detected [72]. Anaerobic bacteraemia has been demonstrated to be always or frequently present in the prolonged TTP group [73, 75]. Indeed, 6/15 (40 %) bloodstream infections that were detected after more than 48 h of culture incubation corresponded to anaerobes [72].

All of these facts – the negative urine culture, the short TTP (3 h) for bottle 1 (* E. coli* and *C. ramosum *) and an immunocompromised elderly patient with abdominal symptoms – led us to suspect fatal *E. coli* bacteraemia.

*C. ramosum* was susceptible to ampicillin, piperacillin/tazobactam, cefoxitin, imipenem, clindamycin and metronidazole. However, it is important to take into account that this species can produce β-lactamases and can display variable susceptibility to other antibiotics, such as penicillin, cefoxitin, quinolones, clindamycin and cephalosporins [3, 18, 41, 76–78, 28, 44]. Carbapenem resistance has also been reported [28].

Treatment of patients with *C. ramosum* infections should combine antimicrobial agents with an early and aggresive elimination of infectiuos foci (drainage, debridement, removal)[19, 21, 28, 32, 34, 35, 39, 344, 46, 47, 48, 49].

## Conclusions

To the best of our knowledge, this is the second case of CRB in an elderly patient with fever, abdominal pain, bilious emesis and fatal outcome. We presumed an intra-abdominal source of bacteraemia, based on the clinical and the polymicrobial presentation with an intestinal inhabitant. For elderly bedridden patients with a history ofrheumatoid arthritis, long-term steroid treatment and new-onset fever, CRB may be fatal with devastating consequences despite adequate surgical and medical management. However, in this patient it was not really proven that *C. ramosum* was the most important pathogen in the sepsis.

Its incidence is probably underestimated because *Clostridium* spp. observed as gram-negative thin rods is fairly uncommon and may be unrecognized from polymicrobial bacteraemia. The interpretation and meticulous observation of Gram stain and proper processing of blood cultures were very important in the bacteriological diagnosis of CRB. MALDI-TOF MS was a fast and reliable methodology for the identification of *C. ramosum *.
